# Sovereignty of what and for whom? The political mobilisation of sovereignty claims by the Italian Lega and Fratelli d’Italia

**DOI:** 10.1057/s41295-022-00273-w

**Published:** 2022-03-28

**Authors:** Linda Basile, Rossella Borri

**Affiliations:** 1 Department of Social, Political and Cognitive Sciences, University of Siena, Siena, Italy; 2 Department of Social, Political and Cognitive Sciences, University of Siena, Siena, Italy

**Keywords:** Sovereignty, Sovereignism, Populist parties, Populist radical right parties, Public opinion

## Abstract

This article looks at the relationship between conflicts of sovereignty and patterns of national party competition, by focusing on the electoral support for two Italian populist radical right parties (PRRPs), the Lega (*the League*, Lega) and Fratelli d’Italia (*Brothers of Italy*, FdI). Using public opinion data, the study finds that the conflicts of sovereignty represent a distinct and multidimensional set of attitudes related to voting preferences. Overall, these conflicts seem to provide some electoral advantage to the PRRPs over other competing parties in the electoral arena. However, they do not provide the same amount of gains to all PRRPs, since ideologies and party identities are important intervening factors in the relationship between conflicts of sovereignty, party mobilisation, and voting behaviour.

## Introduction

Conflicts of sovereignty are increasingly divisive and politicised in the contemporary political debate (Brack et al. 2019; Bickerton et al., early view), and they are profoundly shaping the dynamics of political competition. Such conflicts have provided fertile ground especially for the rise of those political parties belonging to the variegated populist group. This group has mobilised them among the electorates, emphasising issues related to the decision-making authority of the nation-states and people’s empowerment against the elites. Results in recent general elections in several European countries, and the 2019 European elections, confirmed the electoral potential of such demands (Basile and Borri 2020; Taggart and Pirro 2021).

Over the last decade, however, these conflicts have also developed in complexity and multidimensionality (Jabko and Luhman 2019), which has opened a wide range of opportunities for political mobilisation along the lines of such tensions. Claims for “taking back control” can be linked to multiple and rival understandings of sovereignty: national as opposed to supranational sovereignty, popular versus parliamentary sovereignty, and even centralised versus decentralised, or federal conceptions of (subnational) sovereignty within the nation-state (*ibid*; Bickerton 2019). Moreover, these conflicts can develop along several issue domains, such as the economy, migration and border control, and the rule of law. This provides parties with a rich “menu of choice” from which to select the sovereigntist claims that align most closely with their broader ideological platform.

From a demand-side perspective, these multiple understandings of sovereignty translate into a broad range of sovereigntist claims available to electorates (Albertazzi and McDonnell 2007, p. 2; Basile and Mazzoleni 2020). Offered with a variegated list of choices by different political actors, voters can choose to support the party whose claims better correspond to their own idea of “taking back control”. This article explores the complexity and multidimensionality of the conflicts of sovereignty in contemporary European politics by looking at the patterns of party competition that occur on the matter of sovereignty itself. By using survey data, we investigate how different understandings of (national, popular, or subnational) sovereignty are likely to mobilise popular support and shape voting behaviour.

We focus on the electoral support for two Italian populist radical right parties (PRRPs), namely the Lega (*the League*) and Fratelli d’Italia (*Brothers of Italy*, FdI). Italy represents an interesting case study. Especially after the general election of 2018, a variety of populist parties have been electorally successful, each of them emphasising different dimensions of sovereignty. Examining two PRRPs allows us to better highlight how different conceptions of sovereignty are likely to allow a differentiation of the patterns of political mobilisation even within the same area of the left–right continuum.

This article has implications for the scholarly understanding of the role of the conflicts of sovereignty in party competition and provides grounds for developing a conceptual framework to explain the relationship between sovereignty claims and votes. Moreover, it contributes to the literature on PRRPs by highlighting how they can differentiate and compete with each other along the lines of a rich “menu of choice” of sovereignty claims. One of the central messages of this paper is that sovereignty conflicts can be conceived of as internal products of party political mobilisation, based on the deployment of sovereignty claims by political actors in line with the opportunities and constraints offered by new and old ideological traditions. This subjective or constructivist dimension to sovereignty conflicts should be an important part of the research agenda in this field.

## Multidimensional conflicts around blurred borders: the sovereigntist “menu of choice”

Claims to restore the full sovereignty of specific actors and levels of government are not new to political debate. They have been used to call for a country’s resistance to international norms (Spiro 2000), to support the independence of subnational units from the nation-state (Whitaker 1999), or to question the meaning of political representation and citizens’ participation in democratic systems (Habermas 1997; Garsten 2009). In their contemporary version, these conflicts subsume such variety of understandings under the common denominator of the reaction against the ongoing transformations triggered by the processes of globalisation and supranational integration, which have occurred since the aftermath of World War II. As Sassen (1996, p. 11) argues, what is at stake in the contemporary debates on sovereignty is not the definitive demise of the nation-state, but, rather, its dispersion along “a multiplicity of institutional arenas”. This new form of “diffuse sovereignty” has upended the traditional understanding of sovereignty, based upon the principle of “mutually exclusive territories” and the modern theory of the liberal democratic state as based on the popular will of the people within the nation-state (*ibid*; Basile and Mazzoleni 2020). In Europe, these conflicts have been magnified by the processes of European integration, which encouraged the emergence of a *multi-level governance* in which nation-states have progressively shifted (and shared) powers and competencies away, both below and above the state (Marks et al. 1996; Hooghe and Marks 2001; Bickerton et al., early view).

These transformations require a profound “rethinking” of democratic institutions if governance is to remain effective (Held and McGrew 1993). However, recent critical junctures like the sovereign debt crisis of 2008, the migration crisis of 2015 and the COVID-19 pandemic have laid bare the weaknesses of such “rethinking”, by questioning the effectiveness of the shared sovereignty regime to address these crises (Cotta and Isernia 2020; Wolff and Ladi 2020). Supranational institutions have struggled to respond to these crises (Falkner 2016), leading to growing feelings of dissatisfaction and lack of trust towards them on the part of citizens, especially in those countries hit hardest by the crises (Hobolt and Tilley 2014; Drakos et al. 2019; Conti and Marangoni 2020). Moreover, at the domestic level, they have contributed to the erosion of the representativeness of mainstream political parties, increasingly perceived as detached from the electorate (Mair 2013).

A sovereigntist counter-narrative has emerged out of these processes of global transformation. This narrative promises the return to an older, yet more reassuring, distribution of power, one that is rescaled back to the national, or in some cases even subnational, level. Sovereigntist discourses appeal to citizens by suggesting that the solution to global challenges lies in the restoration of sovereignty within thicker and closed borders. So the sovereigntist argument runs, a narrower and more identifiable territorial dimension would allow people to better understand, and control, the decision-making processes, as compared to the current dispersion of powers across territorial levels (Agnew 2009, 2019; Kallis 2018).

The contemporary formulation of the sovereigntist discourse, as earlier argued, develops around a complex and multidimensional narrative, which touches upon three dimensions (Bickerton et al., early view). First, there is the “foundational” (or normative) conflict between the national and the supranational sovereignty. The sovereigntist counter-narrative firmly locates final authority in popular sovereignty, inveighing against the threat of supranational authorities. Second, there is an institutional dimension, which highlights tensions between the citizens and the elected parliaments, the latter often struggling to control executives and to constrain technocratic regional institutions perceived as distant, unresponsive and non-accountable. Finally, there is a third conflict between unitary, or centralised, and more decentralised conceptions of sovereignty, which builds upon the territorial dimension of sovereignty conflicts. Despite the apparent contradiction of demands for greater powers for subnational levels in response to growing global interconnectedness, these claims are reflective of the wider transformations of the sovereign states themselves (Tierney 2005). Indeed, in current liberal democracies, states might find advantageous to “divide the tasks” and share responsibilities with lower levels to tackle increasingly complex decisions (Duchacek 1987; Hooghe et al. 2010).

Within this framework, there are two different sovereigntist counter-narratives to these transformations *within* the states. On the one hand, there is the reassertion of a unitary, national sovereignty through a recentralisation of competencies to the nation-state against centrifugal tendencies from the peripheral regions demanding greater autonomy, if not full independence (Bickerton 2019; Keating 2021). On the other hand, nationalist and regionalist sovereignty claims can be reconciled by the identification of a common enemy outside the subnational and national borders, such as EU institutions or migrants (Mazzoleni and Ruzza 2019).

To add further complexity, conflicts of sovereignty and associated sovereigntist counter-narratives develop along different policy domains, such as socio-economic policy, migration and border control, and democracy and the rule of law. According to such a multidimensional perspective, claims to “take back control” can be conceived either in economic terms, as the recovery of full authority over the national economic policy, or in cultural and identity terms, as the primacy of national culture and identity, or in institutional terms, as the need to increase legitimacy and accountability in decision-making (Brack et al. 2019).

## PRRPs go sovereigntist

Populist parties, both from the left and the right, are the principal beneficiaries of the mobilising potential of contemporary conflicts of sovereignty. Sovereignty conflicts are closely related to the very essence of the populist phenomenon, namely its people-centrism. Populism is indeed a “thin ideology” which conceives of society as a struggle between the two homogeneous groups of “the pure people” and “the corrupt elites” and “argues that politics should be an expression of the *volonté générale* (general will) of the people” (Mudde 2004, p. 543). Drawing on this divide, populist parties claim their role as the only legitimate actors to speak on behalf of the people and, by acting in their name, to claim to take back control of (nation’s, regions’, popular) authority. The chameleonic nature of populism and its ideological “thinness” (Taggart 2004, p. 275; Mudde 2004) allows populist parties to combine such basic people-centrism with a variety of ideological traits (Basile et al. 2020; Stanley 2008), as well as to emphasise different policy issues in their political platform. They can easily strategically adapt their people-centrism to conflicts of sovereignty, by readdressing their antagonistic claims against the supranational, the national or even the subnational elites (Mudde 2004; Basile and Mazzoleni 2020), as well as across different issue domains (the political, the economic, or the cultural domain).

Although the sovereignty claims are strategically modelled by populist actors from both sides of the political spectrum, they assume a distinctive connotation within the heterogeneous group of PRRPs (Borri and Verzichelli 2021). This is linked to the peculiar nature of these type of parties, which combine the anti-élite populist narrative with nativist, exclusionary conceptions of national sovereignty and authoritarian values, both distinctive traits of the far-right (Mudde 2007). This results into a peculiar formula of sovereignty claims, able to channel people’s anxieties and fears into resentments towards different kind of outgroups (Bonikowski et al. 2019).

It is worth noting how PRRPs heavily rely on nativism, which advances a conception of the nation that merges both the ethnic and state nationalism and that “strives for the congruence of the cultural and the political unit, i.e. the nation and the state” (Mudde 2007, p. 16; Bonikowski et al. 2019). They use the populist strategic repertoire that depicts the elites as responsible of most of the sources of contemporary insecurity, from not defending the interests of the national economy against the competition of the international markets and allowing the uncontrolled flows of migrants, to operating within technocratic mechanisms. Accordingly, PRRPs reframe their nationalist arguments into claims for the reappropriation of popular sovereignty against unresponsive elites (Aslanidis 2018; Betz 1994).

Reframing of ethnic and state nationalism into a “people vs. elite” antagonism allows some flexibility in the political discourse of PRRPs, including in the way sovereignty claims are deployed. For instance, these parties can develop a dual frame where nationalist and regionalist claims coexist, as in the case of the Italian Lega (see below) and the Swiss Lega dei Ticinesi (Mazzoleni and Ruzza 2019). This strategy is likely to work when PRRPs shift the “we vs. them” antagonism towards a vertical dimension, so that the two apparently incompatible ethnic communities, the (majority) national and the subnational ones, are reconciled through the identification of a common enemy to both *demoi*, such as the EU or the unresponsive national elites.

## “Keep your friends close, but your enemies closer”: the mobilisation of the conflicts of sovereignty by the Lega and FdI

The ability of PRRPs to combine different sovereignty claims translates into a variety of political discourses used in electoral competition. From a demand-side perspective, this means that different types of voters, who are likely to support different aspects of the sovereigntist counter-narrative, are offered several, tailor-made political proposals from which they can choose.

The capacity to appeal to multiple segments of society is a crucial factor when, in a party system, there are more political actors competing for the same pool of voters. However, we expect that not all PRRPs have the same degree of flexibility in shaping and successfully conveying sovereigntist messages to different audiences. Such an expectation is linked to the fact that populism is a “thin ideology” that sits alongside “fuller” ones (Mudde 2004; Stanley 2008). Populism can be more or less peripheral in defining the identity of a party, depending on the constraints imposed by the deep-seated right-wing radicalism that also defines these parties. We expect that those populist parties with deepest roots in the far-right political tradition would tend to stick to a purely nationalist, exclusionary sovereigntist discourse. This should be reflected in an electorate that is receptive to an exclusive conception of the people as well as to claims concerning the empowerment of the nation-state against the external threats, mainly embodied by migrants, seen as challengers of the national culture and people’s safeness. By contrast, parties with less consolidated far-right roots would be able to combine different understandings of sovereignty, across several issue domains, beyond the migration policies. Moreover, their ideological flexibility would allow them to combine with a single political discourse appeals to different groups of people, such as the national or subnational groups.

To examine how the combination of the populist and the radical right narratives influences the contemporary political debate on the conflicts of sovereignty and, consequently, shape the dynamics of party competition, we focus on the electoral constituencies of two Italian parties, namely the *Lega* and *Fratelli d’Italia*. They represent an interesting case study, since they are both referred to as belonging to the PRRPs group (Rovny and Polk 2020; Taggart and Pirro 2021; Zulianello 2020). They share similar political stances and have electoral alliances at both the national and local levels. They are thus likely to appeal to similar electorates, especially those attracted by sovereigntist counter-narratives, and whose voters could potentially shift from one party to the other (*Expectation 1*).

At the same time, these parties are two outstanding examples of different degrees of ideological flexibility. These differences reflect the ideological underpinnings and the historical traditions of these two parties, which in turn translate into contrasting abilities to mobilise voters via rival understandings of sovereignty. We thus posit that the Lega would be more able than the FdI to integrate into its political offer a variety of sovereignty claims, ranging from the national to the subnational, through the popular sovereignty claim, and across several issue domains, like economic and immigration. Conversely, a lower degree of flexibility would make it difficult for the FdI able to capitalise on any conflicts of sovereignty other than the one focusing on a defence of the national state against “outsiders”, particularly migrants. Nonetheless, this ideological consistency would make the FdI a more credible actor than the Lega for voters sharing their ideology (*Expectation 2*). Before delving into the analyses, the remainder of the section offers a short description of differences and similarities between the two parties.

### From “Roma ladrona” to “Italians first”: the ideological flexibility of the Lega

The Lega was founded in 1991 with the name of Lega Nord (*Northern League,* LN) and, since then, it has profoundly influenced Italian politics, being present in representative arenas and by participating to governments not only at the local and regional, but also at national levels. Created as a federation of autonomist movements of northern Italy to represent their economic interests, its ideological platform inevitably developed around demands for greater subnational autonomy. However, over time, the Lega has changed its platform according to shifting political circumstances (Mazzoleni and Ruzza 2018; Albertazzi et al. 2018). The party’s positions have ranged from full independence, especially between 1995 and 1999, to federalism, as in the 1992–1994 period (McDonnell and Vampa 2016; Massetti and Toubeau 2013; Newth 2019) until the recent “sovereigntist turn”, with the decline of emphasis on demands for the empowerment of the northern Italian regions (Albertazzi et al. 2018).

Besides the territorial discourse, when it first appeared in the political scene, the LN/Lega escaped from any classification along the left–right continuum, presenting itself as a post-ideological party (Diamanti 1993). This allowed the party to attract voters from both sides of the ideological spectrum. Nonetheless, over time, the party was also able to develop a “territorial populist” ideological platform (Albertazzi et al. 2018) that combined claims for subnational autonomy with populist, anti-elite arguments. In the Lega’s rhetoric, both the national elites in Rome and the southern citizens represented the “others”, which served to consolidate the Lega’s “we”, namely, the “people from Padania”. This term, “Padania” refers to an “invented” and vague geographic entity of northern Italy approximately coinciding with the Po Valley. It was defined by convergent socio-economic interests but has lacked any real cultural and historical shared identity (Giordano 2000).

When Matteo Salvini became the party’s federal secretary in 2013, the party further radicalised its populist radical right rhetoric, blending economic neo-liberal positions and support for state intervention, xenophobia and anti-immigrant feelings with support to minorities, pro-EU stances with Euroscepticism and anti-globalism (Albertazzi et al. 2018; Mazzoleni and Ruzza 2019). All this whilst maintaining a strong populist connotation.

The removal of the reference to “North” in its name, in 2017, marked a new, nation-wide strategy, in which the Lega has gradually expanded its electoral constituencies by gaining support across the entire peninsula. Rebranded as “Lega for Salvini premier”^1^ or simply Lega, the current party’s core slogan of “Italians first” has replaced the rhetorical attacks against Rome and the southerners. With the new course, however, the Lega has not replaced but *combined* subnational demands with nationalist claims (Mazzoleni and Ruzza 2019). The current Lega’s statute replaced the reference to the independence of Padania with the “peaceful transformation of the Italian State into a modern federal state” (art. 1) among its main goals. Behind such apparent contradictions there are underlying intra-party tensions stemming from this “rebranding” (Newth 2019), as well as proof of a certain ideological flexibility that allows the party to adapt its political discourse to different constituencies. In a recent interview with Salvini, this adaptability was referred to as the “many costumes” of the Lega leader—from “Fireman Salvini” and “Law and Order Salvini” to his most recent move towards supporting the pro-EU government of Mario Draghi (Johnson 2021).

### A “new” populist party with a long right-wing tradition: the rightist sovereigntism of FdI

Giorgia Meloni’s FdI was founded in 2012 as a splinter from the *Popolo delle Libertà* (People of Freedom). Despite its recent creation, FdI has a much more rooted political tradition and a more marked ideological profile than the Lega. The party traces its origins back in the post-fascist *Movimento Sociale Italiano* (Italian Social Movement, MSI), which later became *Alleanza Nazionale* (AN), and it still keeps these parties’ symbol—the tricolour flame (Borri and Verzichelli 2021). Besides the symbols, the FdI inherited from these parties a strong radical right discourse, which prioritises issues related to national identity, Italian culture, and traditional values. Accordingly, although FdI shares the populist rhetoric with the Lega, based on the anti-elite appeals to popular sovereignty, the party’s claims to “take back the control” are mostly related to typical radical right cultural and identity aspects, such as immigration, the alleged process of “Islamisation”, or same-sex marriages. Likewise, the restoration of the nation-state control is mostly expression of the opposition to the EU’s multicultural model, described as a threat to the national values and identity. Such a deep-seated political tradition constrains this party’s ability to adjust the party’s discourse to changing circumstances and audiences in the way the Lega does. However, this articulation of the conflicts of sovereignty identity and cultural divides makes FdI a more credible player when people feel that their way of life is threatened by far-reaching societal changes.

### Data and variables

For our empirical analyses, we rely on a subset of 2,599 Italian citizens aged 18 years and older from the broader IMAJINE survey,^2^ conducted between 22 September and 15 October 2020. The sample was recruited from an opt-in panel provided by the survey company Toluna, following a quota sampling procedure based on gender, age, and geographical macro area.^3^ The questionnaire was administered using a CAWI (Computer Assisted Web Interview) methodology. The dataset includes weights to correct coverage biases in non-probability sampling (Battaglia et al. 2009; Baker et al. 2013; Valliant and Dever 2018). Further technical information on the survey, survey questions and full descriptive analyses of the variables included in the models are presented in Appendix available from the authors upon request.

#### Dependent variable

Our dependent variable is the voting intention for Lega and FdI, measured with the question on voting preference asking which party people are more likely to vote for, in case a General Election would take place tomorrow. Within the sample, 15% of respondents expressed preference for the Lega, while 11% were likely to vote FdI.

#### Independent variables: conflicts of sovereignty

To measure the *Economic and Borders’ sovereignty*, we used a set of questions asking respondents’ propensity to retain full state-control over decision-making, as opposed to the supranational authority (i.e. the EU) on economic and budgetary policy (*Economic sovereignty*), and immigration policy (*Borders’ sovereignty*). *Popular sovereignty* is measured by the question that asks people to choose between a system where decisions are made by elected politicians and one where ordinary people make all decisions on their own. Finally, to measure *Subnational sovereignty* we used two variables: the first one provides respondents’ support for different degrees of division of competencies between the central state and the subnational authority (i.e. regions in Italy), namely centralisation, decentralisation, and federalism; the second one asks people’s support for a subnational authority’s decision to become independent, which represents the fullest expression of subnational sovereignty. All variables were recoded into a 0–1 scale, such that higher values indicate support for sovereigntist claims.

#### Control variables

The model also includes a set of control variables, selected among those factors that have been found to be key to explain support for PRRPs, or that are closely related to the conflicts of sovereignty. First, we consider attitudes on the issues related to perceived threats to the nation (Lubbers and Coenders 2017), such as the *immigration threat* (Betz 1994; Ivarsflaten 2008; Golder 2016), and the *European Union* (Arzheimer 2018), which are found to be the main drivers of radical right-wing voting. As for the latter, since the nationalist backlash might translate into a support for leaving the European Union (van Kessel et al. 2020), we use a variable measuring *people’s propensity to leave the European Union*.

To take into account the feelings of belonging to and identification with the nation, which are inherent to the conflicts of sovereignty, we include a measure of *national identity*. This has also has been found to be and additional explanation of the vote for radical right parties (Lubbers and Coenders 2017). By the same token, since our analysis looks also at the subnational sovereignty, we consider *subnational identity* as a potential source of support for parties defending subnational sovereignty. The two levels of national and subnational identification are not necessarily incompatible and mutually exclusive, however. In multi-layered contexts like Europe, they are likely to be nested and complementary, with people often feeling attached to both their subnational region and the nation at the same time (Medrano and Gutiérrez 2001; Moreno 2006). Accordingly, our national and subnational identity variables precisely measure the extent to which individuals feel, respectively, more attached to the nation than the EU and to the subnational level than the nation.

The variables on *trust in national and European institutions* have been included because they are related to arguments on popular sovereignty and, at the same time, a widespread distrust in the political institutions has been considered as a common feature of PRRPs’ supporters (Schulte-Cloos and Leininger 2021; Mudde 2007; Betz 1994). In order to control for voters’ ideological predispositions, we used, besides the *self-placement on the left–right continuum,* also other measures that better capture left–right (economic) positions, such as the *attitudes towards redistribution* (Kleider and Stoeckel 2019) and preferences for *state versus market* economic policies. Scholarly literature has traditionally highlighted the ability of radical right parties to combine welfare chauvinist appeals based on the promise of redistribution of resources reserved to natives (Goubin and Hooge 2021), with pro-market pleas, opposition to high taxation, and a too heavy bureaucracy (Röth et al. 2018; Rovny 2013; Kitschelt 1995).

Socio-demographic controls look at the impact of *gender, age, education, occupation,* and the *migration status*. For instance, men (Spierings and Zaslove 2017), young individuals, and people with low education (Hooghe and Marks 2018) are considered as likely categories of radical right supporters, although some studies have found only a moderate role of these variables in explaining right-wing vote (Stockemer et al. 2018). On the other hand, working class members would represent a large share of the right-wing electorate (Spies 2013), although this might be due more to cultural protectionism, and namely the defence of the national identity against “the others”, than to economic grievances (Goubin and Hooge 2021; Oesch 2008). Finally, the variable on the *migration’s status* (internal, external migrant or no migrant) was included to ascertain whether a person’s mobility has an impact in moderating the feelings of exclusiveness related to the nation (Lubbers and Coenders 2017), which, on turn, would drive votes towards the right wing. All models control for the effect of the *geographical macro area*, to consider the main Italy’s geopolitical divisions.^4^

## Results

We conducted multinomial logistical analyses to assess, first, whether the conflicts of sovereignty drive vote towards Lega or FdI, as compared with other parties; second, whether each of these conflicts provides a different amount of electoral advantage to each of them.

**Fig. 1:**
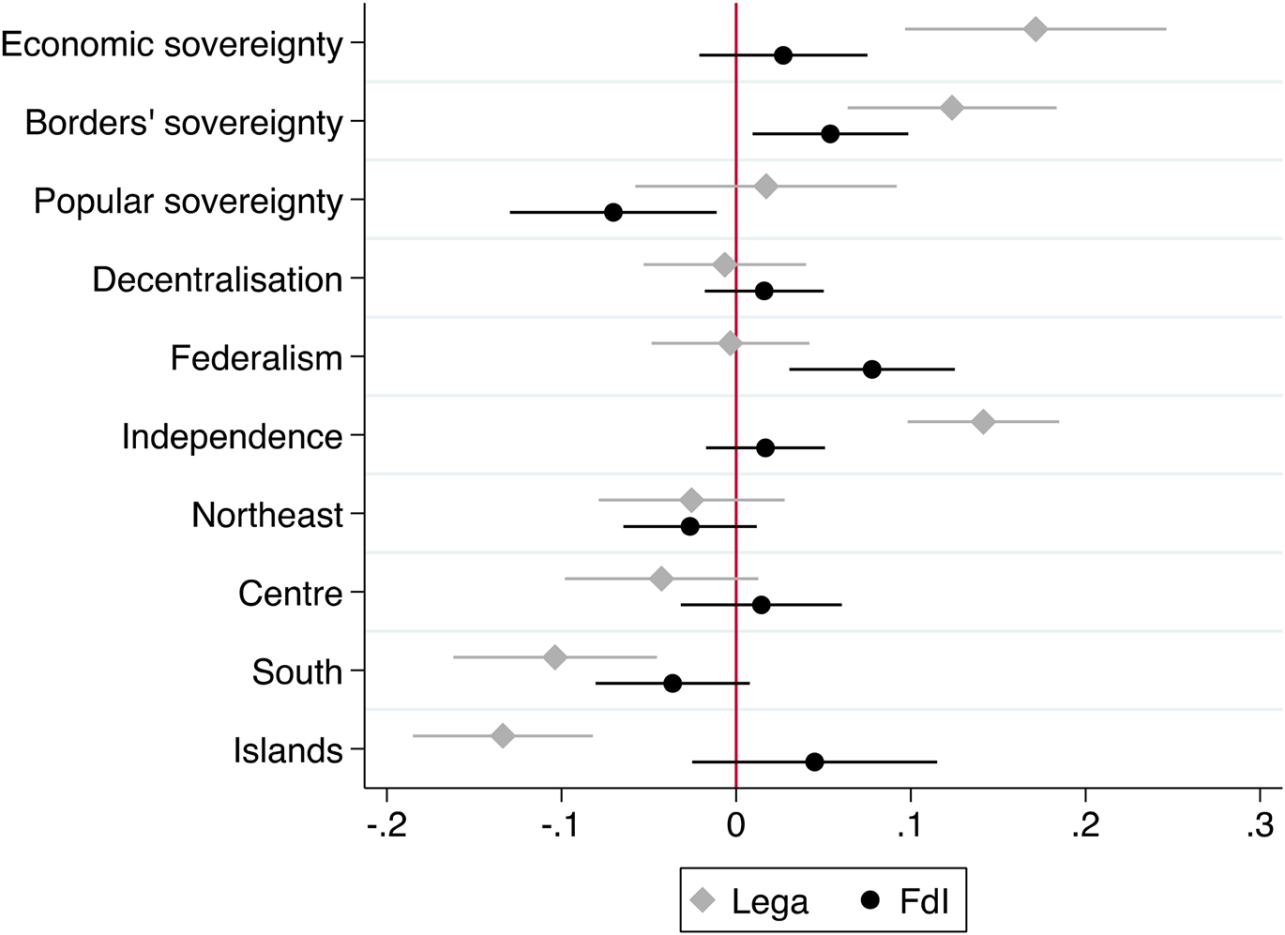
Average Marginal Effects (AMEs) of Attitudes towards conflicts of sovereignty on voting for Lega and FdI

**Fig. 2:**
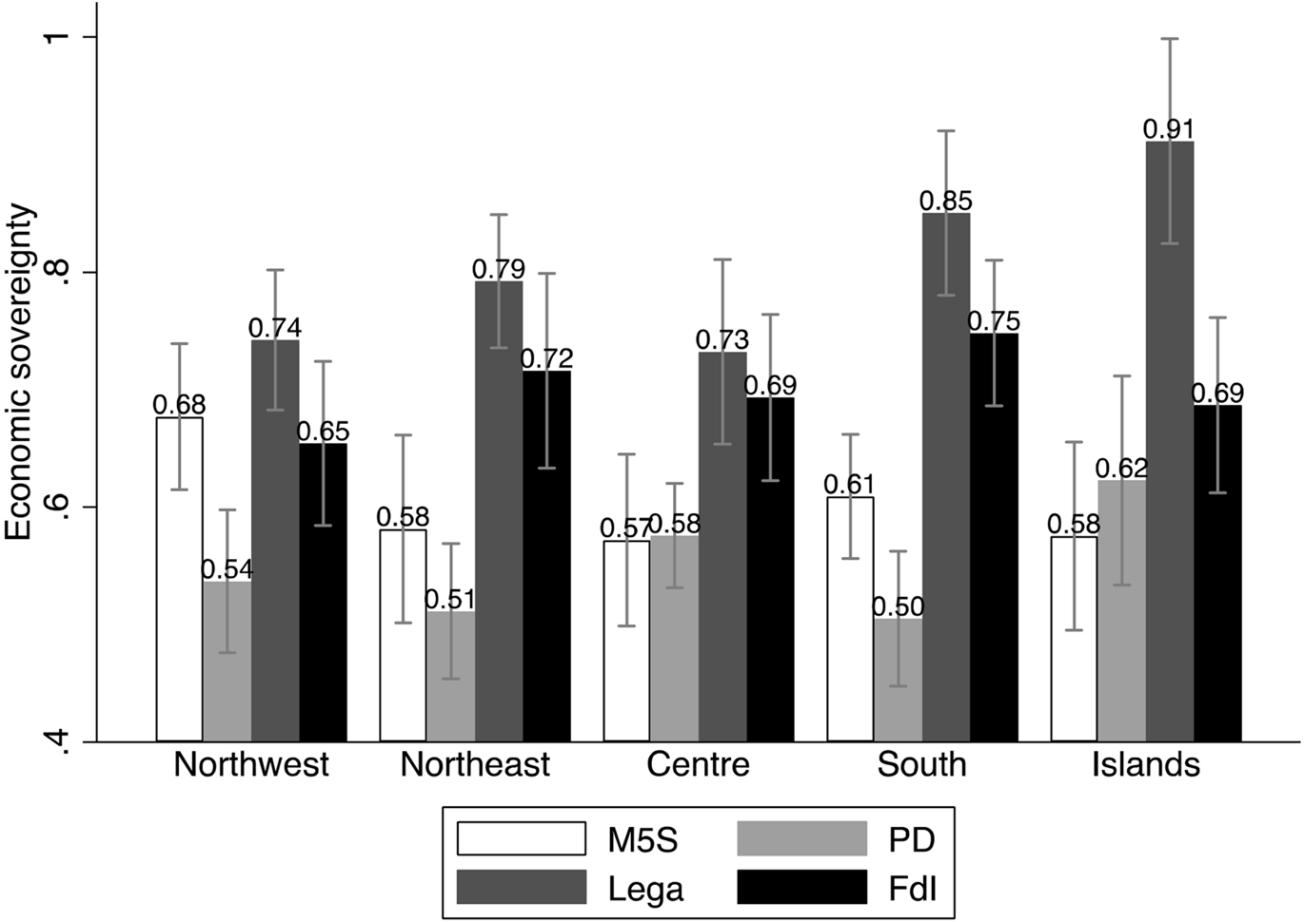
Economic sovereignty and vote (means and confidence intervals)

The first two models include only the main predictors, and they have, respectively, all other parties, and FdI, as base outcome. We use the graphical representations of the Average Marginal Effects (AMEs, Fig. 1), as well as bivariate analyses (Figs. 2 through 6), to help interpreting the models’ coefficients (reported in Appendix). For readability purposes, the figures comparing the Lega and FdI with other parties display results only for the populist *Movimento 5 Stelle* (Five Stars Movement, M5S), and the centre-left party *Partito Democratico* (Democratic Party, PD).

**Fig. 3:**
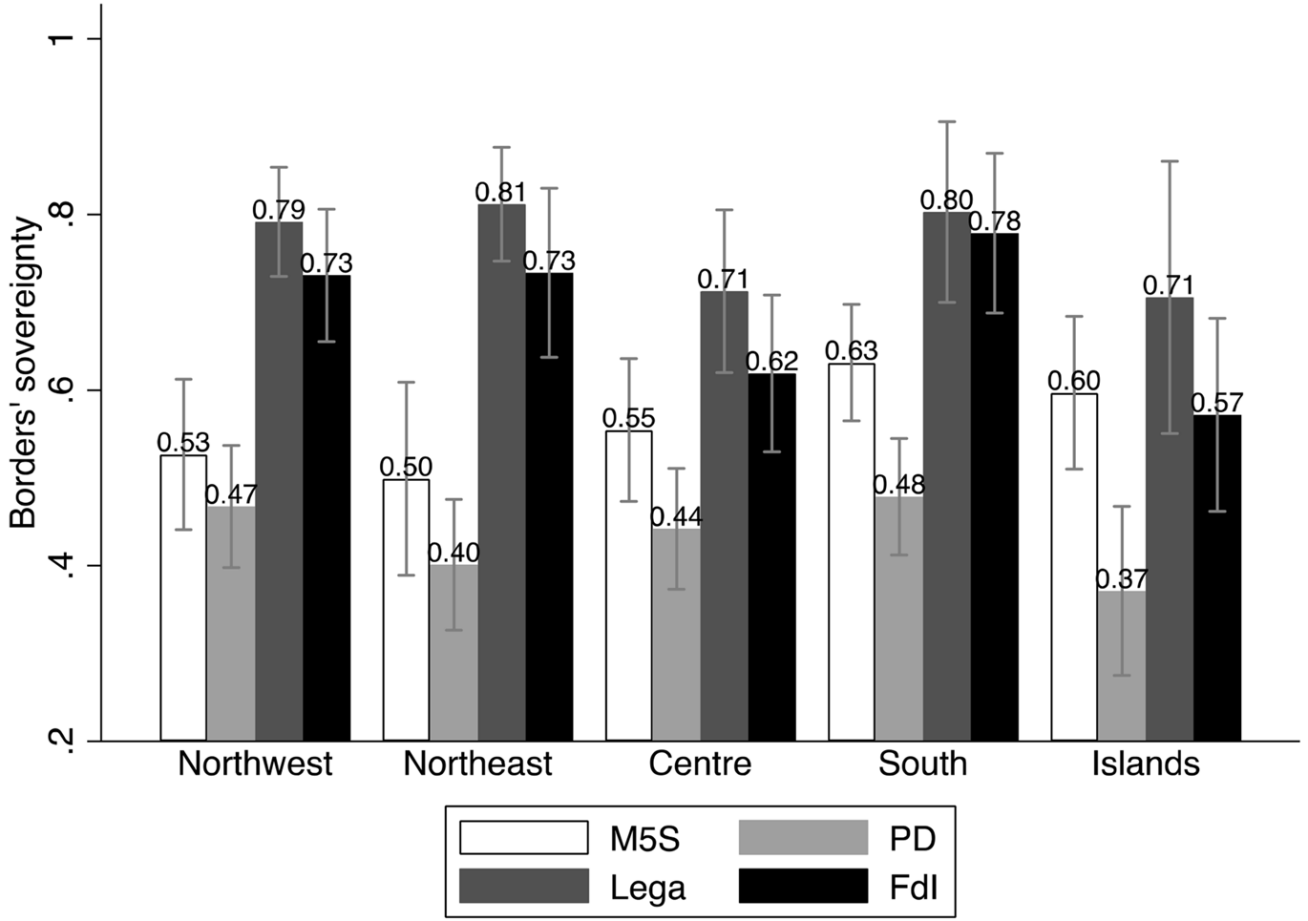
Borders’ sovereignty and vote (means and confidence intervals)

These baseline models suggest that claims to restore *economic sovereignty* (Fig. 2) significantly attract voters to the Lega and only to a lesser extent to FdI, with the Lega being favoured over FdI. Likewise, claims to restore the *sovereignty of borders* (Fig. 3) mark a significant difference between both the Lega and FdI’s electorates and other voting options, although the effect seems to represent a greater source of electoral advantage for the Lega as compared to FdI. Moreover, both types of conflicts play a significant role in driving vote to the Lega across the whole country. Conversely, FdI gets only a moderate support from voters who are high on the economic sovereignty dimension in north-western regions, where it competes with other parties like M5S. The same occurs with borders’ sovereignty in the Islands, where FdI competes with other actors like the M5S on this dimension of conflict, and with the Lega in the other regions.

**Fig. 4:**
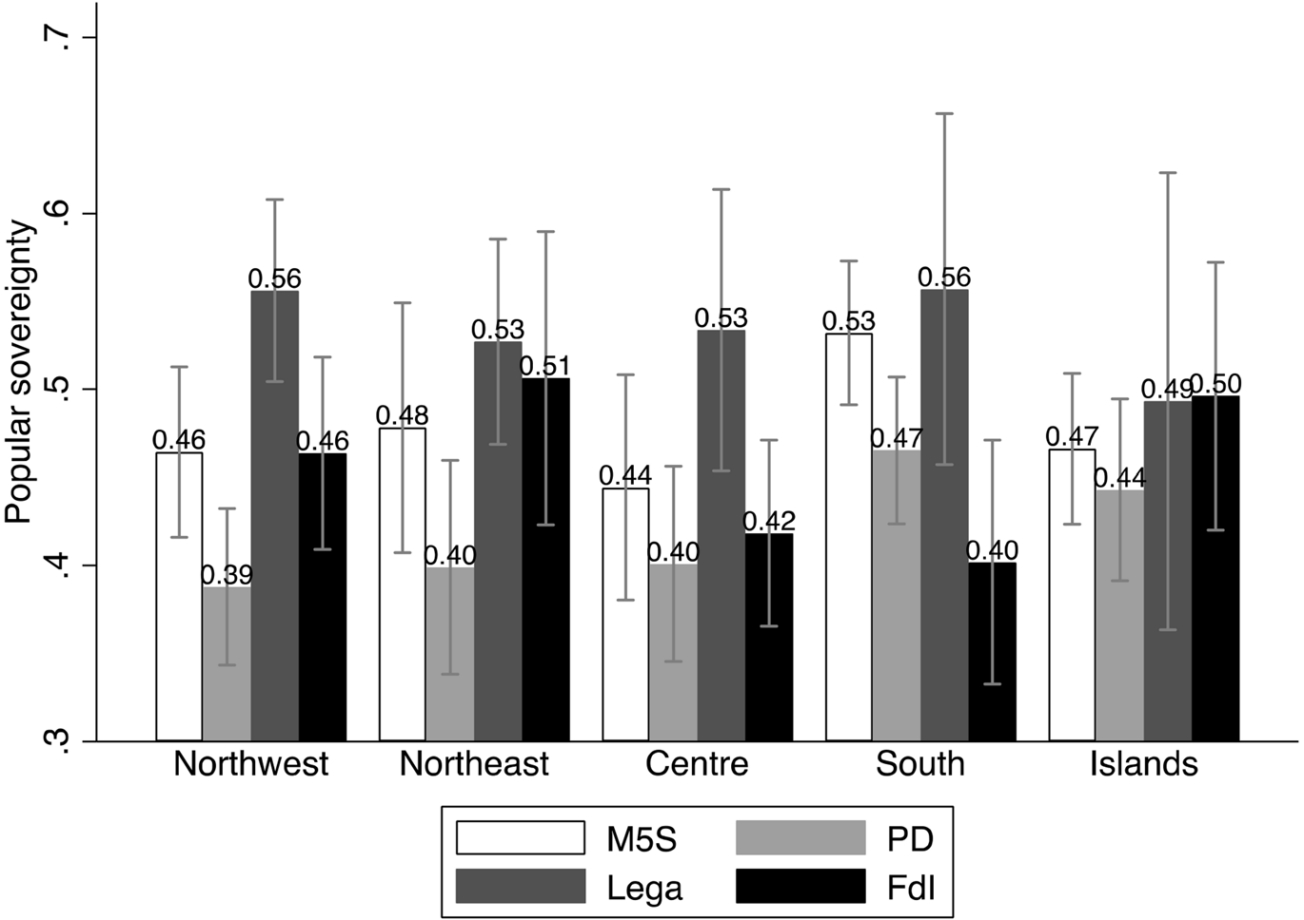
Popular sovereignty and vote (means and confidence intervals)

Popular sovereignty, on the other hand, plays only a moderate, yet not significant role, in favour of the Lega, while it even discourages voting for FdI almost everywhere but in the Islands (Fig. 4). On this dimension of conflict, the Lega competes directly with the other populist actors, the M5S, especially in southern regions.

**Fig. 5:**
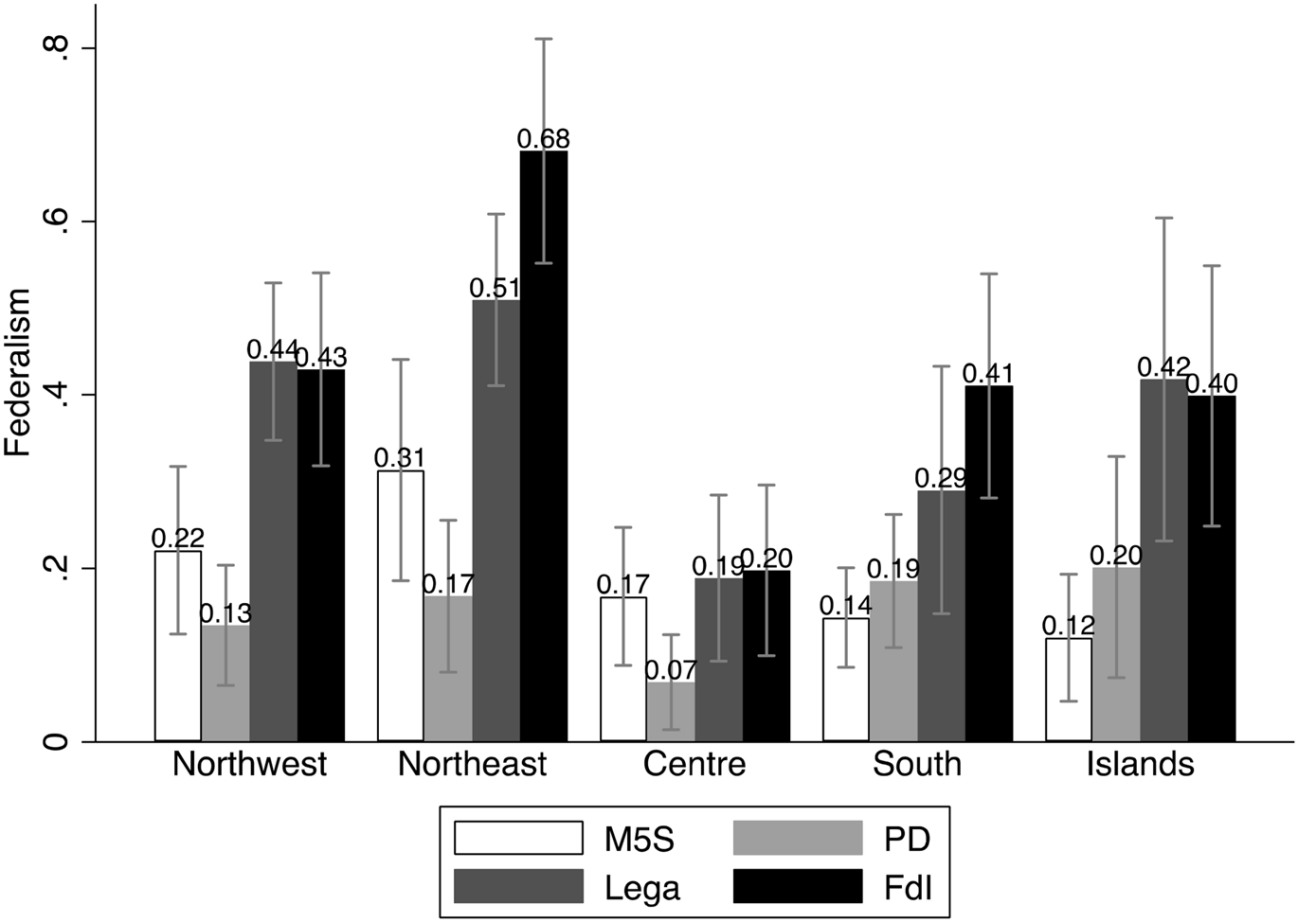
Support for federalism and vote (means and confidence intervals)

**Fig. 6:**
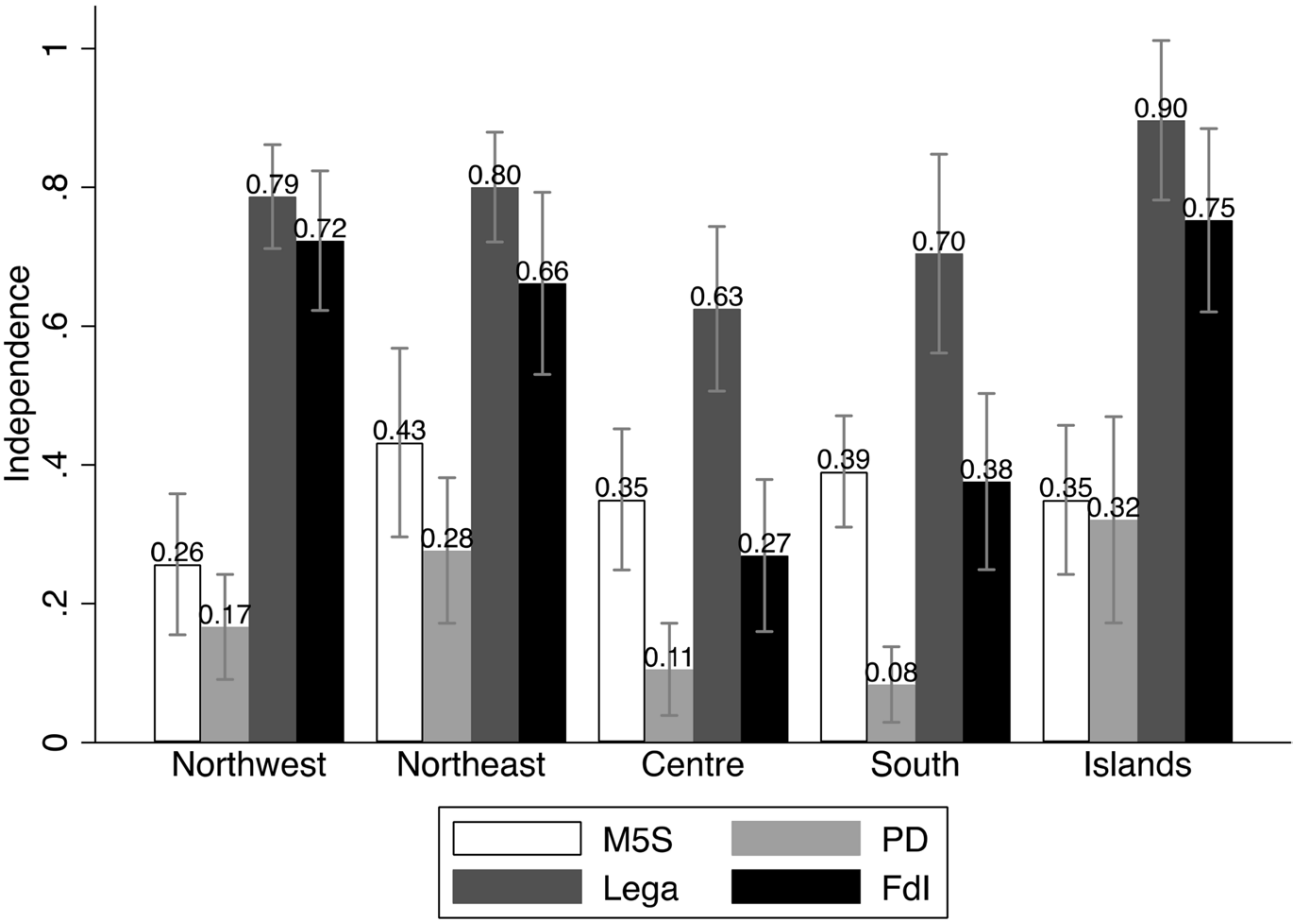
Support for independence and vote (means and confidence intervals)

Looking at the *subnational sovereignty*, support for some decentralisation has not a significant effect on voting for neither the Lega nor FdI, although it comparatively drives more votes in favour of FdI than the Lega. Interestingly, supporters of federalism seem to be diffused in both electorates, but they are more likely to prefer the FdI over the Lega, especially in the Northeast and the South (Fig. 5). Not surprisingly, however, independence claims attract significantly more voters to the Lega than to any other party, across all regions, although there is a relevant share of supporters of regions’ independence also within the FdI’s electorate in northern and central regions (Fig. 6).

### The full model

**Fig. 7:**
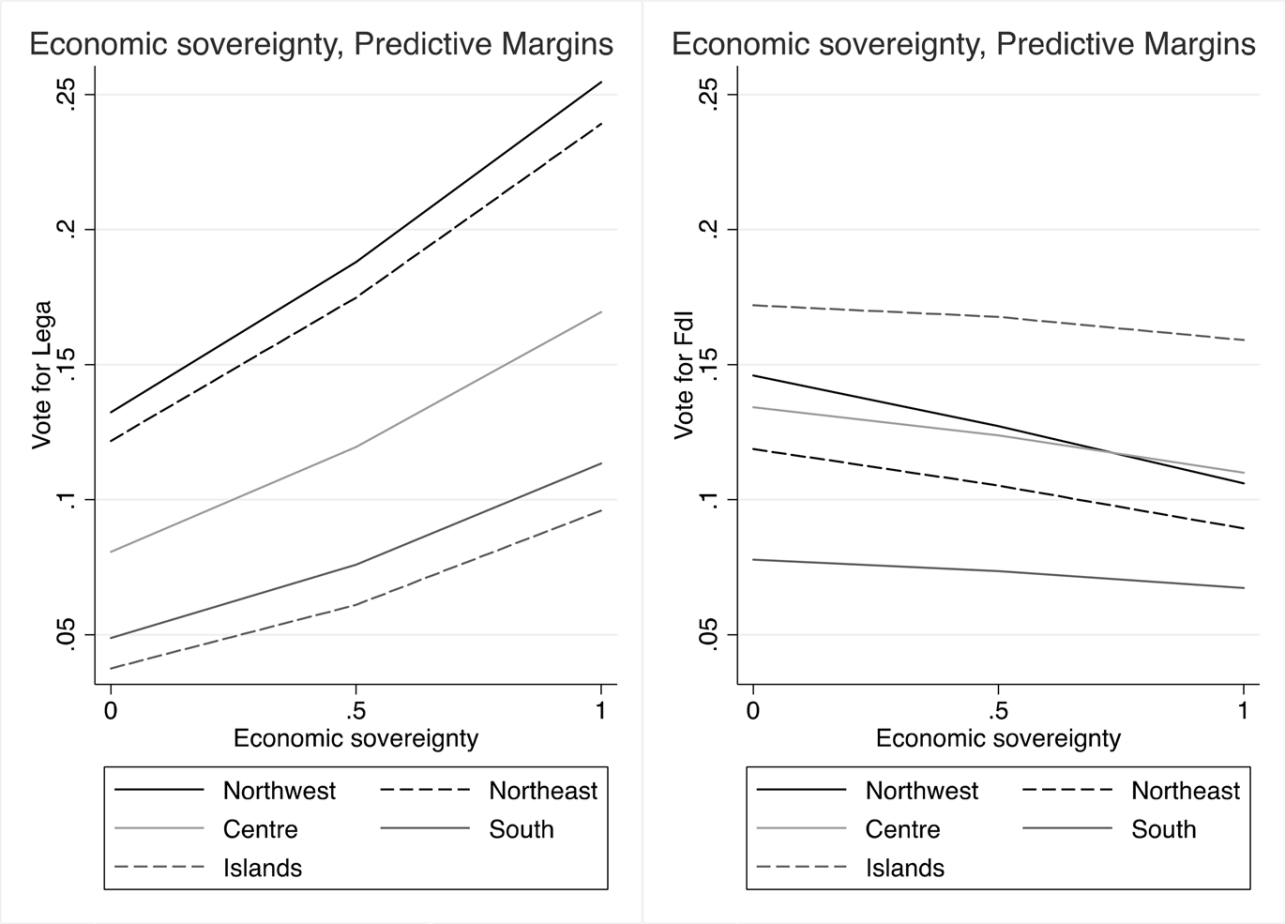
Predicted probabilities of voting Lega and FdI by attitudes on economic sovereignty

**Fig. 8:**
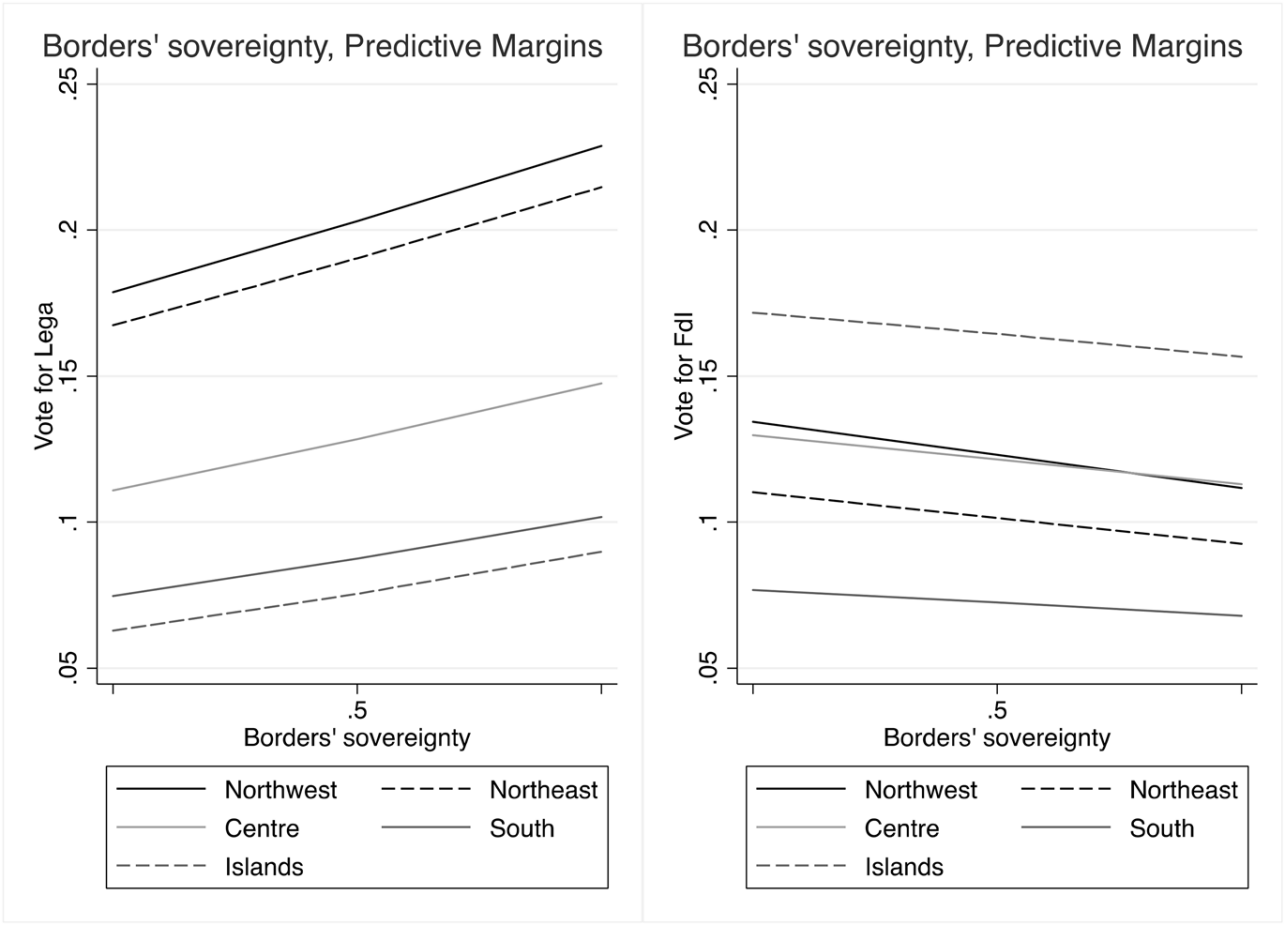
Predicted probabilities of voting Lega and FdI by attitudes on borders’ sovereignty

**Fig. 9:**
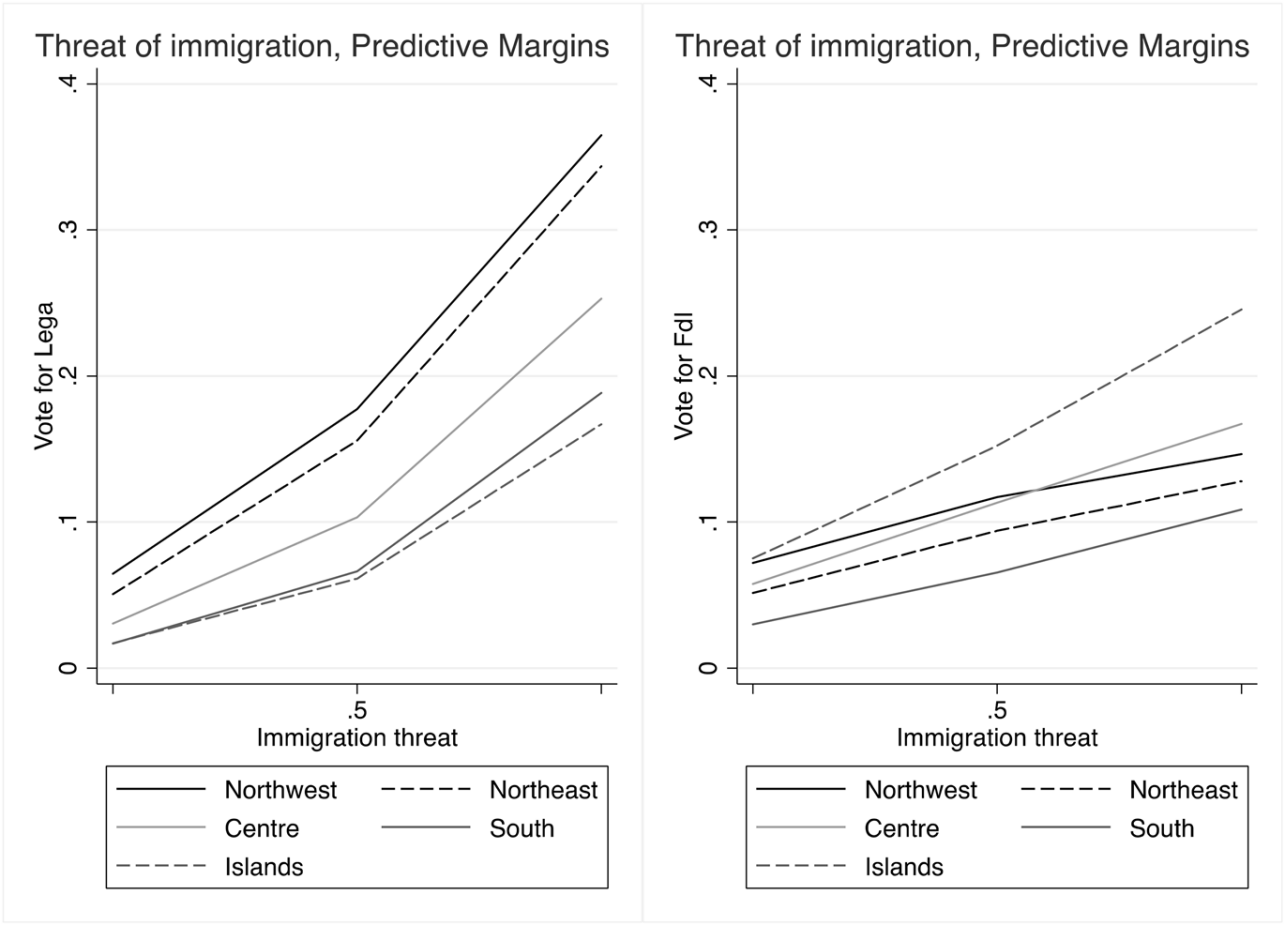
Predicted probabilities of voting Lega and FdI by attitudes on immigration (1 means highest perception of threat)

**Fig. 10:**
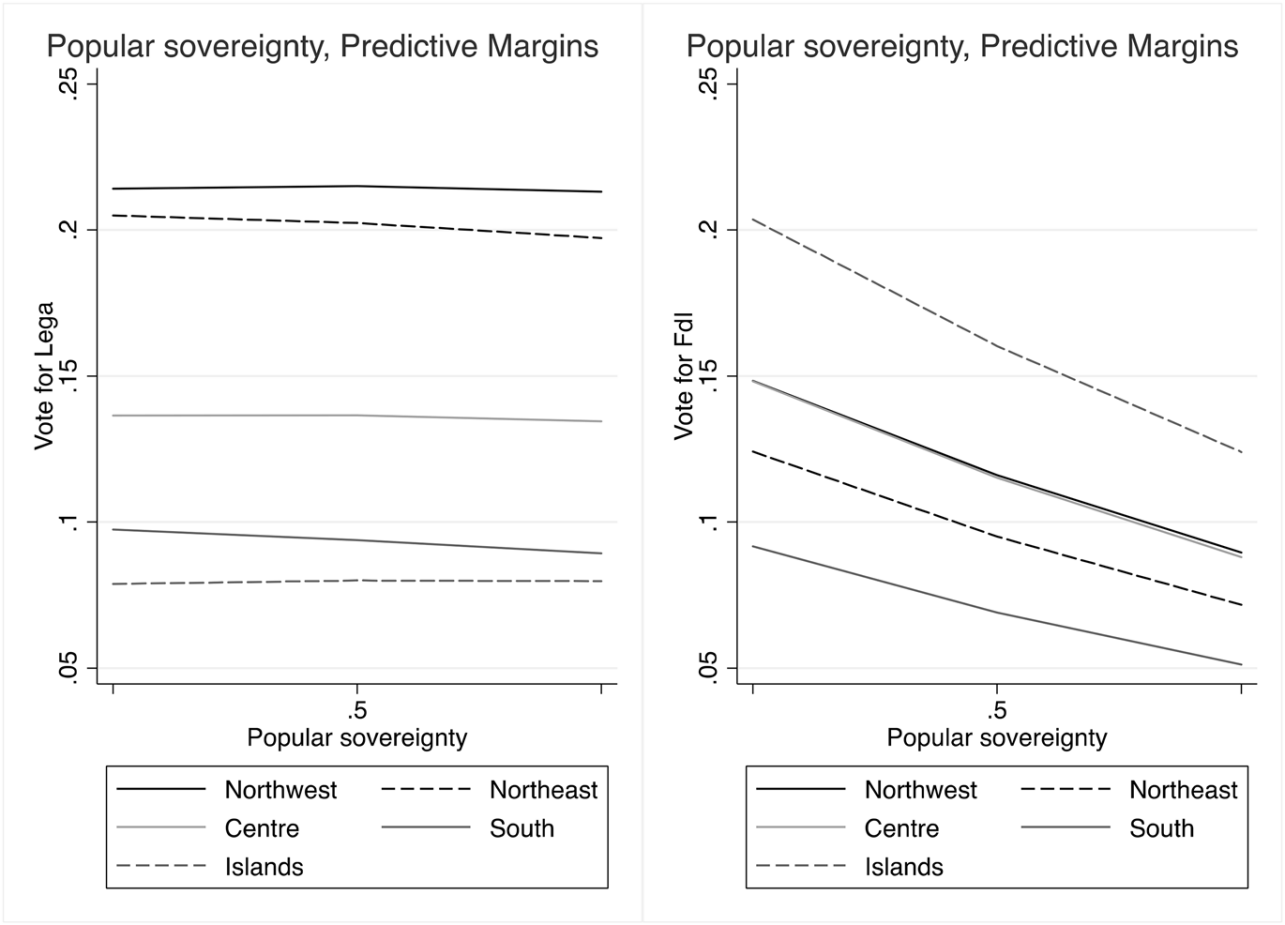
Predicted probabilities of voting Lega and FdI by attitudes on popular sovereignty

**Fig. 11:**
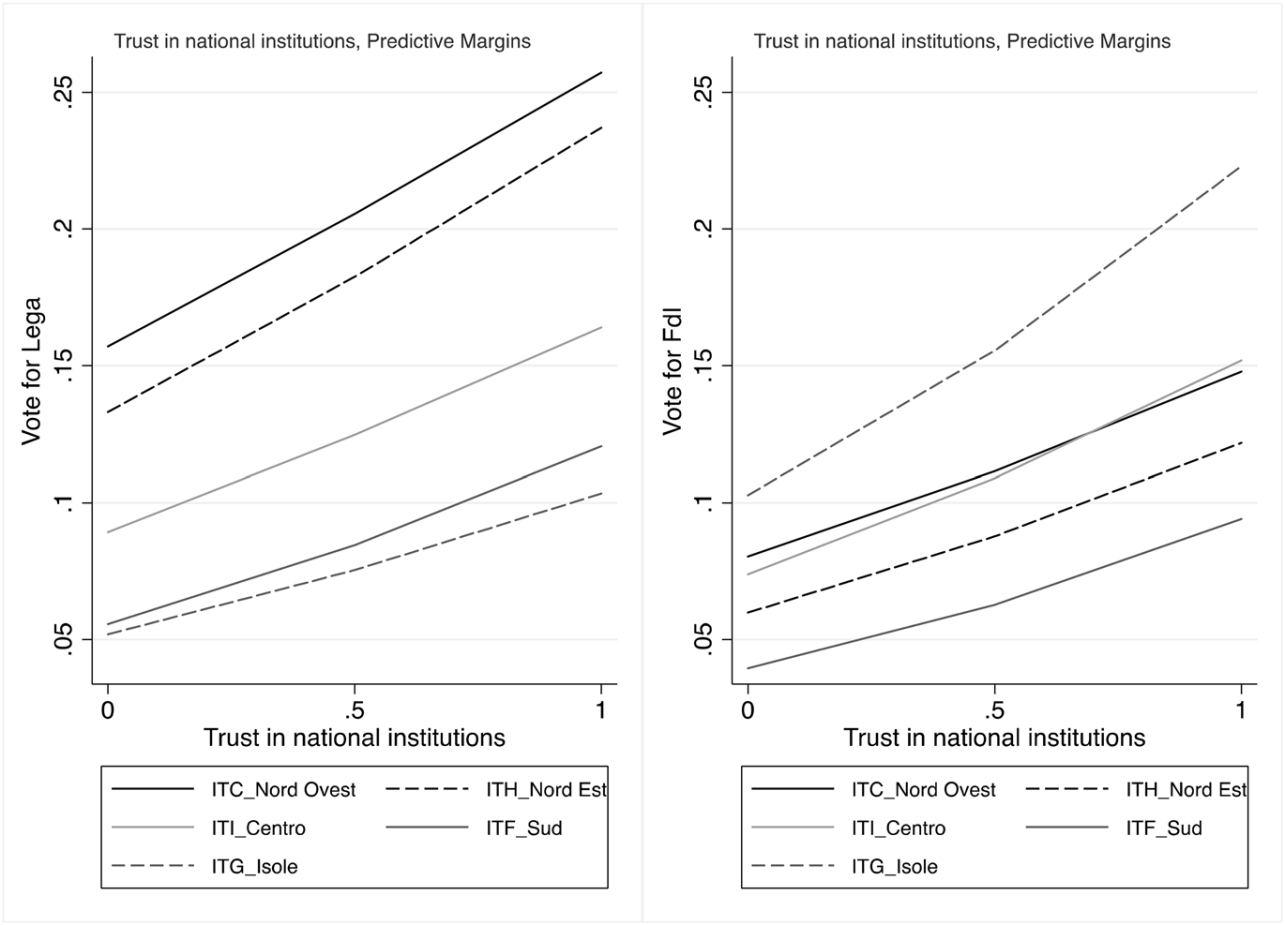
Predicted probabilities of voting Lega and FdI by trust (1 means low trust)

**Fig. 12:**
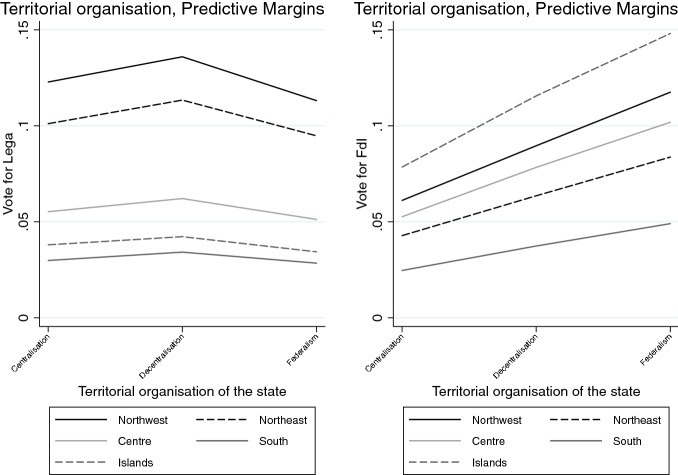
Predicted probabilities of voting Lega and FdI by attitudes on territorial organisation

**Fig. 13:**
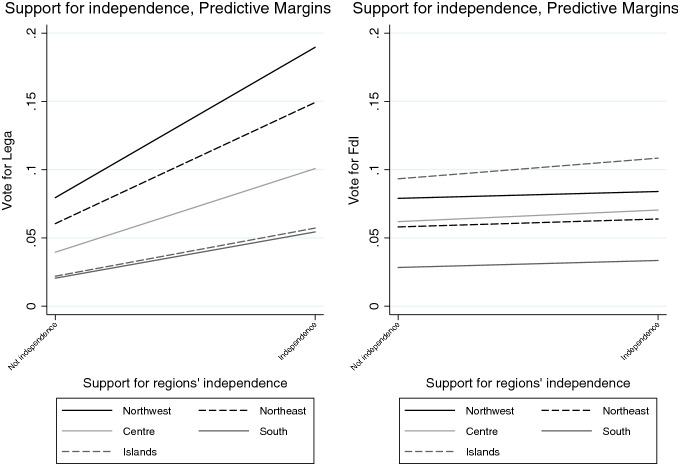
Predicted probabilities of voting Lega and FdI by support for subnational independence

We integrate the models with additional control variables, to sort out rival hypotheses. In particular, we want to assess whether people’s attitudes about conflicts of sovereignty have an independent effect on voting for the Lega and FdI or if they are just a side aspect of other common explanatory factors of voting for PRRPs. To further inspect data, we plot predicted probabilities of voting for Lega and FdI by levels of support for claims of sovereignty, over the five Italy’s geographical macro areas (Figs. 7–13, the full coefficients are reported in the Appendix).

Figure 7 confirms the role of *economic sovereignty* on voting behaviour, with an increase of the probabilities of voting for the Lega, across all regions, and especially in the north of the country. Conversely, it has no or decreasing effect (in northern regions) on voting for FdI. Unlike the baseline models, the full models reveal instead that claims to control national borders (*sovereignty of borders*) have only a modest and not significant effect on voting for the Lega, while it even shows a declining effect on voting for FdI (Fig. 8). In parallel, however, we observe that the probabilities of voting Lega decidedly increase with fear of immigrants, and, even if less markedly, also for FdI, especially in southern regions and in the Islands (Fig. 9).

The analyses confirm that conflicts of popular sovereignty do not represent an electoral advantage for both the Lega and FdI, and it has even a modest, although not significant negative effect on voting for FdI (Fig. 10). We also note, however, that distrust in national institutions enhances the probability of voting for the Lega or FdI over other parties (Fig. 11) everywhere in the country.

Finally, after controlling for other variables, the probability of voting for the Lega decreases among those who support a federal state, while the probability of voting for FdI increases, with a more pronounced effect in northern regions (Fig. 12). Conversely, across all Italy’s subnational regions, and especially the northern ones, the perspective of independence increases the probability to attract votes to the Lega, while it has no significant effect on voting for this party (Fig. 13). The models also suggest that holding a strong subnational identity plays a significant role in explaining votes for the Lega as compared to the other parties, although it does not make a difference in voters’ choice between the Lega and FdI.

Besides the perception of immigration, trust in institution and regional identities, the other attitudinal control variables (i.e. support for leaving the EU, trust in the European institution, national identity, state vs. market) have no significant effect on voting for Lega and FdI. Only the preferences for welfare redistribution seem to be a potential source of electoral advantage for FdI over the other voting alternatives. Likewise, socio-demographic variables have no or only a modest effect on votes. Finally, as expected, both parties significantly attract more voters from the right, but also those who place themselves in the centre tend to choose Lega or FdI, over other voting options.

## Discussion

Our analyses corroborate the argument that conflicts of sovereignty represent a distinct and multidimensional set of attitudes, which are related to voting preferences. Overall, these conflicts seem to provide some electoral advantage to the PRRPs over other actors (*Expectation 1*), even after controlling for other variables. This finding defies the argument that the claims to restore economic sovereignty are just an expression of nationalism or simply another form of Euroscepticism, or that support for subnational sovereignty is merely another way to frame subnational identity.

The first exception to our *Expectation 1* is represented by the conflicts over the sovereignty of borders. Indeed, when controlling for other factors such as the perception of the immigration threat, the state’s control of borders plays no significant role in shaping voting preferences. This result confirms the pivotal role of anti-immigrant feelings in driving the vote for PRRPs and suggests that conflicts over the sovereignty of borders is principally another way of framing anti-immigration policies. The claim to take back the control of a nation’s borders, in this sense, would be instrumental to the defence of the nation against outsiders.

The second, partial exception is represented by the conflicts of popular sovereignty. A closer inspection at the data, using the comparison of coefficients between different outcomes of the dependent variable, reveals that this dimension of conflict significantly drives votes towards other parties, like the M5S, although it has limited, when not negative impact on PRRPs. An apparent contradiction, however, emerges when noting in parallel that a low trust in national institutions encourages voting for those PRRPs that claim to restore the decision-making authority of national institutions. Nonetheless, as earlier argued, PRRPs are able to merge the nationalist claims to “defend the nation” with harsh criticisms of ruling elites. Accordingly, those voters who are disenchanted by how the politics work and, at the same time, think that the politicians are not doing enough to “defend the nation”, look at the radical populist right as a reliable and trustworthy alternative to the current national ruling elites.^5^ These (rightist) voters do not seem to be attracted to more inclusive and deliberative models of decision-making based on popular active participation, as posited by the supporters of popular sovereignty.

Another result emerging from our study, in line with our *Expectation 2*, is that these conflicts do not provide the same amount of potential electoral advantage to *all* PRRPs. We found the ideological flexibility of the Lega as potentially more prone to include a variety of conflicts of sovereignty in its discourse than FdI. At the same time, differences in the electoral support for these two parties by levels of sovereignty are likely to be due also to geographical differences. For instance, the Lega is particularly able to mobilise electoral support in the northern constituencies on claims supporting economic sovereignty, in line with its traditional appeal among the entrepreneurial sectors in the productive regions of the north of the country. The ideological constraints of the FdI, with a deep-seated radical right-wing origin, make it able to capitalise electoral support on the traditional rightist issues, and especially the immigration threat. In this case, it appears as a more credible actor than the Lega, at least in certain geographical contexts like the Islands—which are at the forefront of the migration flows in the country.

The finding concerning *subnational sovereignty* only apparently contradicts this last argument. Our data, in fact, showed that those individuals who favour a federal structure of the state are likely to vote for FdI, despite its centralist tradition—it is indeed the heir of the MSI that has been a fierce opponent of the decentralist reforms in Italy (Basile 2019).

However, it should be noted that Italy has undergone over the last 25 years a profound process of decentralisation, which culminated in the constitutional reform of 2001. The resulting quasi-federal territorial structure, although paving the way to institutional tensions, has become familiar to Italians. This has made regional autonomy and federalism by far a less contentious issue of party competition, even among parties that have traditionally opposed decentralist reforms (*ibid*). Consequently, the supporters of federalism can plausibly rely on a party with a former unitary and centralist tradition to pursue the perspective of stronger sovereignty for their own region. At the same time, the long tradition of electoral and government alliances between the LN and the FdI’s predecessor AN, which have followed with a few interruptions since 1994, have not only implied compromises and agreements with the coalition partners, and especially AN, on decentralising reforms, but also a reciprocal influence on the attention to subnational autonomy.^6^

When it comes to more radical alternatives like subnational regions’ independence, the Lega still appears as the main credible actor, especially in the electoral strongholds of the North, where the independentist demands remain relevant. For instance, the Lega’s President of the Veneto Luca Zaia has been outspoken in his requests for greater autonomy for the Veneto region. The consultative referendum held in Lombardy and Veneto in 2017 to ask for an enhancement of autonomy confirmed such diffused support for subnational sovereignty in the North. The electoral expansion of the Lega well beyond the North has required the party to reduce its emphasis on traditional Lega’s territorial demands like federalism or independence (Mazzoleni and Ruzza 2019; Albertazzi et al. 2018), in favour of other issues like migration control and welfare chauvinism to attract voters in the centre and southern constituencies. This may have weakened Lega’s credibility on subnational autonomy, especially in its traditional electoral strongholds, which would account for why a large share of federalist supporters of the North looks at FdI as a likely alternative to the Lega.

## Conclusions

By looking at how people’s attitudes towards sovereignty conflicts shape voting behaviour in the cases of Italy’s Lega and the FdI, this paper contributes to the broader debate around new conflicts of sovereignty in at least four respects. First, the findings show how the transformations of sovereignty and the crisis of party democracy have profound consequences for the dynamics of political competition and electoral mobilisation. Second, analyses revealed that some conflicts of sovereignty represent distinct sets of attitudes, which are likely to provide electoral advantages to specific types of parties, such as the PRRPs. However, and this is the third paper’s key finding, these conflicts do not provide the same amount of gains to all PRRPs, since parties have to face the opportunities and constraints provided by existing ideologies and identities when they deploy conflicts of sovereignty in their political discourse. Finally, exploring the differences and similarities between two PRRPs, which seem to attract distinct electorates across a variety of understandings of sovereignty, help us to think of these conflicts as the source of multiple sovereignty claims, a sort of “menu of choice” occurring at different levels of governance and in relation to different policy areas.

## Footnotes

1 The “Lega for Salvini Premier’” was created in 2017 as parallel movement to the Lega Nord, to represent the party outside its northern constituency. Since 2019, the Lega manages the entire membership recruitment, with no geographical distinction, while the former Lega Nord still formally exists mainly for judicial reasons, to repay nearly €49 million in state financing (Reuters 2019).

2 The survey was conducted as part of the IMAJINE project, funded by the European Commission under the H2020 program (Grant Agreement n. 726950). The survey was carried out in eight European countries (Italy, France, Germany, the Netherlands, Poland, Romania, Spain, and the UK).

3 In Italy, the sample size was defined at NUTS 2 with about 170 respondents for each subnational unit, although the target could not be achieved in some smaller regions.

4 The macro areas have been identified according to the NUTS 1(Nomenclature of Territorial Units Statistics) subdivision: ITC (Northwest), ITH (Northeast), ITI (Centre), ITF (South), and ITG (Islands).

5 It should be noted that at the time when the survey was conducted both the Lega and FdI were at the opposition. This might contribute to the perception of the Lega as alternative actor to the ruling national institutions, together with FdI.

6 This argument was also confirmed during a semi-structured interview with Mr. Roberto Maroni of the Lega that was conducted as part of the task of the IMAJINE’s project, which analyses party strategies of regionalist actors like the Lega: “[there was a] contamination effect of the LN, as AN has developed a greater attention towards the territories” (Online interview, 6th April 2020).
